# Tackling Microbial Contamination in Polydioxanone-Based Membranes for Regenerative Therapy: Bioengineering an Antibiotic-Loaded Platform

**DOI:** 10.1021/acsabm.5c00263

**Published:** 2025-04-30

**Authors:** Victoria L. Abdo, Jamil A. Shibli, Raphael C. Costa, Maria H. Rossy Borges, Ademar Wong, Maria D. P. T. Sotomayor, Martinna Bertolini, Luciene C. Figueiredo, Valentim A. R. Barão, Elidiane C. Rangel, Joao Gabriel S. Souza

**Affiliations:** † Dental Research Division, Universidade Universus Veritas Guarulhos, Guarulhos 07023-070, Brazil; ‡ School of Dentistry, Alfenas Federal University (UNIFAL-MG), Alfenas 37130-001, Brazil; § Departamento of Prosthodontics and Periodontology, Piracicaba Dental School, Universidade Estadual de Campinas (UNICAMP), Piracicaba 13414-903, Brazil; ∥ Institute of Chemistry, São Paulo State University, Araraquara 14801-970, Brazil; ⊥ Department of Periodontics and Preventive Dentistry, University of Pittsburgh School of Dental Medicine, Pittsburgh, Pennsylvania 15213, United States; # Laboratory of Technological Plasmas, Institute of Science and Technology, São Paulo State University (UNESP), Sorocaba 18087-180, Brazil; $ Faculdade Israelita de Ciências da Saúde Albert Einstein, Hospital Israelita Albert Einstein, São Paulo 05653-120, Brazil

**Keywords:** membranes, guided bone regeneration, biofilms, bacteria, antimicrobial properties, amoxicillin

## Abstract

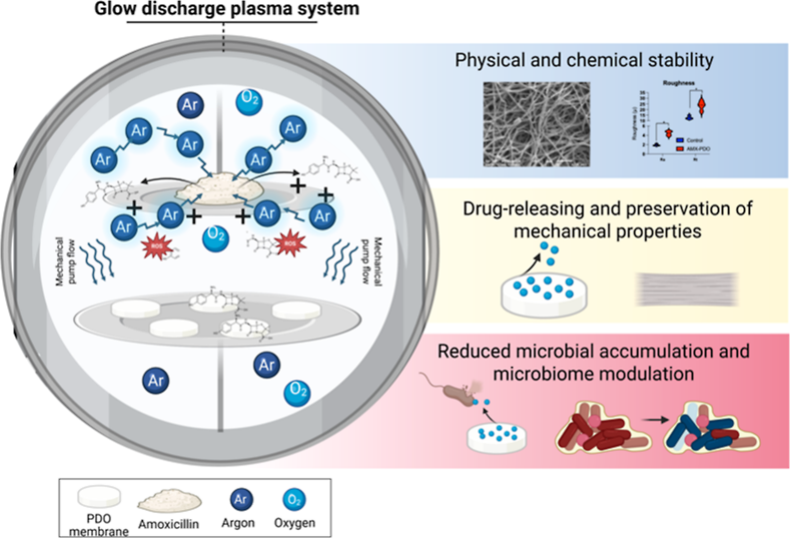

Barrier membranes are essential components of tissue regenerative therapies, acting as physical barriers to protect the healing site. Although collagen-based membranes are widely used, they degrade enzymatically, often triggering inflammation and cytotoxicity arising from residual cross-linking agents. Synthetic polymer-based membranes, such as polydioxanone (PDO), present customizable properties, predictable degradation rates, and induce bone formation more effectively. However, both materials are at risk of exposure to the microbial contamination. To address this, antibiotics have been loaded onto membranes as drug-delivery systems, a strategy that has not yet been explored for PDO membranes. In this study, the oral polymicrobial contamination of PDO-based membranes was evaluated and compared with collagen membranes and aimed to develop an amoxicillin-loaded PDO (AMX-PDO) membrane. For this purpose, PDO membranes with different pore sizes (0.25, 0.50, and 1.00 mm) and two commercially available collagen membranes were evaluated, using in vitro and in situ models, in terms of polymicrobial accumulation. Next, AMX-PDO membranes were developed by glow discharge plasma using Ar and O_2_ gases and an amoxicillin compound. The findings revealed similar microbial levels for both PDO and collagen-based membranes, but PDO membranes modulated microbial composition with reduced (∼3–5 fold-decrease) levels of specific oral pathogens. The AMX-PDO membrane maintained similar physical and chemical properties to those of untreated membranes, but it significantly reduced polymicrobial accumulation and prevented microbial cells from passing through them. Thus, they acted as more than passive physical barriers only, but rather as biologically active barriers. Therefore, amoxicillin loading on PDO barrier membranes by means of plasma technology seems to be a promising strategy to prevent local infection during regenerative therapy.

## 1 Introduction

Human body structures are often affected by tissue damage, resulting in the loss of architecture, structure, and function, as observed in bone defects, which pose a significant clinical challenge.^1^ To address tissue damage, tissue engineering approaches such as guided bone regeneration (GBR) and guided tissue regeneration (GTR) are commonly used in dentistry. The aim of these therapies is to promote healing and restore bone and/or other tissues by re-establishing the lost tissue architecture and facilitating targeted regeneration.^2^ Bone loss due to biofilm-related diseases and tooth loss can compromise the feasibility of oral rehabilitation and the restoration of oral functions.^3^ Bone regeneration around dental implants with previously contaminated threads presents significant challenges, primarily due to the difficulty in achieving effective decontamination of the implant surface.^4^ Contaminated threads harbor biofilms and microbial deposits that compromise tissue integration and may perpetuate inflammation.^5^ Chemical agents such as chlorhexidine, hydrogen peroxide, citric acid or even systemic and local antibiotics are applied to reduce the microbial load further and enhance surface biocompatibility.^5,6^ Despite these efforts, complete decontamination is rarely guaranteed, thus making the subsequent steps of regeneration even more critical to the overall success of the procedure. Once the implant surface has been decontaminated, GBR is used to restore the lost bone and stabilize the implant.^7^ During GBR, to protect graft materials and promote bone regeneration, barrier membranes are commonly used as passive physical barriers to prevent invasion of nontarget tissues, such as epithelial or connective tissue, and microbial cells, while allowing regenerative cells to populate the defect area.^8^

In clinical settings, various types of membranes have been used, including nonresorbable and resorbable materials, which can be used alone or in combination with bone substitutes and/or growth factors.^9^ An effective barrier membrane should facilitate the transport of blood, nutrients and protein-rich body fluids to the bone defect area, while maintaining a pore structure that retains cell products.^8,10^ Resorbable membranes have an important advantage, they do not require a re-entry surgery for membrane removal.^11^ Among them, collagen-based membranes are the most commonly used for GBR due to their high biocompatibility, biodegradability, vascularization, their capacity for inducing cell migration arising, and low immunogenicity.^12^ However, collagen membranes often have unfavorable mechanical properties, such as low rigidity and poor mechanical strength.^13^ Although cross-linking methods have been applied to enhance the mechanical properties of collagen-based membranes, these techniques may reduce biocompatibility, due to potential cytotoxic effects arising from residual cross-linking agents.^14^ In recent decades, efforts have been made to produce collagen-based materials blended with other polymers, such as chitosan, poly-*l*-lactide (PLLA), and hyaluronic acid, to enhance biological responses, promote cell differentiation, and mimic human tissues.^15,16^ However, these methods can also lead to changes in membrane degradation rates, reduced swelling behavior, and decreased collagen content, which is directly dependent on the polymer used in the blend. Furthermore, clinical evidence is still limited.^17^ To improve the mechanical properties of resorbable membranes, synthetic polymers have been introduced in the market over the past few years, and have gained prominence in regenerative therapies due to their customizable mechanical properties, predictable degradation rates, and structural versatility. Synthetic polymer-based membranes offer significant advantages for GBR, such as the capacity for good space-maintenance, a slow resorption rate, adaptability to bone defects, and enhanced biodegradability, which have led to promising clinical outcomes.^18,19^ Among them, the polydioxanone (PDO)-based membranes have demonstrated higher levels of induction of bone formation and osteogenic differentiation when compared with collagen-based membranes,^20^ with the advantage of undergoing hydrolytic degradation, and being broken down into nontoxic byproducts (primarily glycolic acid).

Despite their popularity, both type of membranes can often become exposed to the oral environment, which can lead to microbial contamination, triggering inflammatory responses, and potentially compromising wound healing.^21^ It is noteworthy that the impact of polymicrobial contamination on these membranes has not been experimentally tested. Barrier membranes can be colonized by oral pathogens through exposure to the oral environment or contact with microbial-rich oral fluids.^21^ This allows microbial cells and their metabolites to migrate to the wound healing site, potentially triggering an inflammatory process that affects bone formation and even degrading the membrane structure.^21,22^ Therefore, researchers have proposed that a local drug delivery system could help prevent microbial challenges during the healing process in regenerative therapies, with different antimicrobial agents being applied to or incorporated into membrane materials.^21^ However, the majority of methods have resulted in short-term drug release which has provided insufficient time for microbial control postoperatively, or they have often compromised the physical and mechanical properties, and biocompatibility of the membrane.^21,23^ Although systemic antibiotics have been widely used in clinical practice, they may lead to microbial resistance and reduced effectiveness at the infection site.^24^ Therefore, loading antimicrobial agents onto barrier membranes for local delivery has been widely explored, but has rarely been successful. Amoxicillin, the most used antibiotic for oral infections, has been loaded onto membranes, but its antimicrobial effects have typically been tested on specific microbial species^25,26^ without considering the polymicrobial nature of oral infections, or its functionalization on PDO-based membranes.

Therefore, the aim of this study was to characterize the polymicrobial contamination of PDO-based membranes using in vitro and in situ models, and compare them with collagen-based membranes. An additional aim was to develop amoxicillin-loaded PDO (AMX-PDO) membranes by using glow discharge plasma technology, to control microbial accumulation while simultaneously preserving the biological, physical, and mechanical properties of the barrier membranes.

## 2 Materials and Methods

### 2.1 Materials and Substrates

Commercially available PDO-based membranes (Plenum Guide, Plenum Bioengenharia, Jundiai, Brazil) with different pore sizes (0.25, 0.50, and 1.00 mm) were carefully cut into disc shapes (ø = 12 mm × 0.5 mm). These PDO membranes are synthetic materials manufactured by using the melting electrospinning process. Natural double-sided porcine collagen-based membranes, commercially available as Bio-Gide (control 1) (Geistlich Pharma AG, Wolhusen, Switzerland) and Straumann Jason (control 2) (Straumann, Basel, Switzerland), were used as controls. PDO membranes with different pore sizes were compared with collagen-based membranes for microbial contamination. The PDO-0.50 membrane, the size most used in clinical practice, was selected as a substrate for antibiotic loading treatment. Amoxicillin trihydrate (C_16_H_19_N_3_O_5_S·3H_2_O) (Nova Química, Barueri, Brazil) was used for allowing discharge plasma treatment.

### 2.2 In Vitro Polymicrobial Contamination

Commercially available PDO membranes with different pore sizes (PDO-0.25, PDO-0.50, and PDO-1.00) and collagen-based membrane controls (control 1 and 2) were evaluated in terms of polymicrobial colonization using a validated in vitro model for implant-related infections.^27,28^ For this purpose, a pool of fresh stimulated human saliva from five volunteers with good systemic and oral health was used as a polymicrobial inoculum to mimic the human oral microbiome. The inclusion and exclusion criteria for volunteers were described elsewhere.^29^ The study was approved by the Local Research and Ethics Committee (56455222.6.0000.5506) and all participants signed the term of informed consent. Membrane discs were incubated with the salivary microbial inoculum in BHI medium (Becton-Dickinson, Sparks, MD, USA) (10:1 v/v) + sucrose (10:1 v/v) at 37 °C with 5% CO_2_ for 24 h and 72 h. Samples were washed 3x with 0.9% NaCl before analysis to remove detached cells. Experiments were conducted in duplicate. Biofilms were analyzed in terms of live cell counts, microbial composition and biofilm structure.

### 2.3 Microbial Live Cells and Biofilm Composition

After polymicrobial biofilm formation, membrane discs were immersed in microcentrifuge tubes containing a 0.9% NaCl solution and vortexed for 30 s to detach cells. Thus, a serial dilution of a vortexed suspension was plated on Blood Agar plates to estimate live cell counts by colony-forming unit (CFU). Live cell counts were expressed on a logarithmic scale or fold-over. For microbial composition, biofilm suspensions were analyzed by checkerboard DNA–DNA hybridization technique.^30^ This technique allows evaluation of the presence and levels (proportions) of 40 bacterial species highly associated with the progression of oral biofilm-related diseases, such as periodontal disease and implant-related infections.^29,30^

### 2.4 Membrane Morphology and Biofilm Structure

The morphology of PDO and collagen-based membranes was analyzed using scanning electron microscopy (SEM; JEOL JSM-6010LA, Peabody, MA, USA) by means of electron beams with low accelerating voltages (3 kV). Biofilms formed on the membranes were also evaluated for their structure by using SEM imaging. For this purpose, membranes with biofilms were fixed for 2 h in Karnofsky’s fixative solution (containing 2.5% glutaraldehyde, 2% formaldehyde, and 0.1 M sodium phosphate buffer at pH 7.2). Thus, specimens underwent a series of ethanol washes for dehydration. Once dried, the specimens were mounted on stubs, sputter-coated with a thin layer of gold, and subsequently analyzed by SEM operating at 15 kV.^31^

### 2.5 In Situ Biofilm Formation

To evaluate the microbial colonization of PDO membranes with different pore sizes, an in situ study was conducted by exposing the membranes to the oral cavity of five healthy volunteers to microbial accumulation. In clinical practice, since barrier membranes are commonly placed over implantable devices during oral rehabilitation, membrane discs were mounted on titanium (Ti) discs (ϕ12 mm × 2 mm) made of Ti–6Al-4 V powders with a particle size of 25–45 μm, manufactured by means of additive manufacturing technology (Plenum–Jundiai, São Paulo, Brazil). A previously established in situ model was used,^28,29^ in which a palatal appliance containing membrane discs of each pore size (0.25, 0.50, and 1.00) (ϕ = 12 mm × 0.5 mm) were placed over Ti discs. These discs and membranes fixed in the appliances were protected by plastic mesh to allow biofilm accumulation. Samples were exposed extraoral 4×/day to a 20% (v/v) sucrose solution to promote the growth of implant-associated pathogens, in order to achieve bacterial loads similar to those observed in clinical trials involving patients with implant-related infections.^29,32^ On the morning of the fourth day, membranes were collected from volunteers and analyzed for live cell counts. Ti discs not covered by membranes were used as controls for maximum biofilm formation. Membranes not used for live cell counts were analyzed using SEM imaging, similar to the procedures described above.

### 2.6 Antibiotic-Loaded Membrane Development

The antibiotic treatment was conducted by using a plasma-enhanced chemical vapor deposition technique.^33,34^ For this purpose, a PDO membrane with a pore size of 0.50 mm was chosen since it is widely use in clinical practice and because it qualitatively showed fewer adhered bacterial cells when compared with the other pore sizes. The glow discharge system consists of a stainless-steel reactor chamber with two parallel-plate, horizontal, circular electrodes, a radiofrequency (RF) generator operating at 13.56 MHz with an output power of up to 300 W, and a vacuum system.^35^ For the plasma treatment, PDO-0.50 membrane discs were bound onto the upper electrode and the amoxicillin powder (1 g) was dispersed on the lower electrode. Then, the reactor was evacuated to a background pressure of ≈2.0 × 10^–2^ Torr. Plasma depositions were prepared from mixtures of 85% Ar and 15% O_2_ applied using 13.56 MHz and 150 W in the lower electrode for 15 min. The total pressure of gases of 4.0 × 10^–2^ Torr was kept for all treatments. After 900 s of the process, membrane discs were removed from the reactor at room temperature. AMX-PDO-treated membranes were evaluated for their physical, mechanical, biological, and microbiological properties. Untreated PDO-0.50 membranes were used as controls.

#### 2.6.1 Surface Topography/Morphology of AMX-PDO-Treated Membranes

The surface morphology of the treated and control membranes was analyzed using SEM (JEOL JSM-6010LA, JEOL), and their chemical composition was evaluated through energy-dispersive spectroscopy (EDS) with a detector attached to the SEM, operating at beam energies of 5.0 and 10.0 keV.^34^

#### 2.6.2 Molecular Structure

Fourier transform infrared spectroscopy (FTIR) was performed using a Jasco FTIR 410 spectrometer (Tokyo, Japan) to analyze the molecular structure of the AMX-PDO and control membranes. The spectra represented the average of 128 scans acquired at a resolution of 4 cm^–1^.^34^

#### 2.6.3 Membrane Surface Roughness

The surface roughness of the membranes was measured using topographic profiles acquired with a profilometer (Dektak D150; Veeco system). The arithmetic roughness (*R*_a_) and total roughness (*R*_t_) were estimated in micrometers (μm).

#### 2.6.4 Extensional Tensile Resistance

Tensile tests were conducted using a UXF (Universal Extensional Fixture) accessory, where DMA tensile tests were performed. Based on the specified maximum torque, it was possible to calculate the maximum allowable total stress. The tensile tests were conducted to determine the linear elastic region of the material, where the slope of this linear region on the extensional stress vs strain graph represents Young’s modulus. The higher the Young’s modulus, the greater the stress required to produce a certain deformation. Oscillatory measurements were performed under a constant prestress overlay. Extensional Viscosity Test–Multidrive: for extensional stress (σ), the number of points was defined by the equipment (264 points), and a logarithmic ramp profile from 0.01 to 30 s was selected. The extensional strain rate ranged from 0.1 (1/min) to 1 (1/min). A thermostatic bath was used at a temperature between 10 and 15 °C. At the end of the test, the following were estimated: extensional tension, extensional deformation, and Young’s modulus. The AMX-PDO was compared with that of an untreated control membrane.

#### 2.6.5 AMX-PDO-Treated Membrane Degradation

To evaluate the degradability of the membranes, AMX-PDO and control membrane discs were incubated in a 0.9% NaCl solution (2 mL) at 37 °C for 30 days. The membrane weights were measured before the assay and after 30 days to assess weight loss as an indicator of degradability, in accordance with ISO 10993-13:2010 guidelines.

#### 2.6.6 AMX Drug-Release

For the drug release assay, AMX-PDO membranes were placed in tubes containing 0.9% NaCl solution, and the solutions were collected after 1, 3, and 7 days to evaluate amoxicillin concentration using high-performance liquid chromatography with ultraviolet detection (HPLC-UV). The amoxicillin solution was prepared weighing a mass of 3.65 mg of this compound and solubilized in 0.1 mol L^–1^ sodium hydroxide solution in a 5 mL volumetric flask. HPLC-UV analysis was conducted on a Shimadzu model 20A liquid chromatograph coupled to an SPD-20A UV/vis detector, an SIL–20A autosampler and a DGU–20A5 degasser, controlled by a microcomputer. A C18 column (250 × 4.6 mm, Shim–Pack CLC–ODS) was used, positioned inside a Shimadzu CTO–10AS oven to maintain a constant temperature. The total running time for amoxicillin standard solution was 7 min using mobile phase 10^–3^ mol L^–1^ phosphate buffer (pH = 5.0) and acetonitrile in the ratio 95:5, v/v and λ = 229 nm.^36^ Results were expressed in ppm.

#### 2.6.7 Biological Response–Protein Adsorption

Since protein adsorption is considered the first biological response in the human body,^37^ AMX-PDO and control membranes were subjected to protein adsorption assays. A pool of stimulated human saliva from three volunteers was collected at least 2 h after eating and brushing their teeth. The saliva pool was centrifuged (10 min at 3800 g) to remove cell debris and contaminants. Membrane discs were then immersed in 1 mL of saliva in a 24-well plate at 35 °C for 2 h. Subsequently, the membranes were washed three times with 0.9% NaCl to remove nonadsorbed proteins. The membranes were then immersed in NaCl solution, vortexed, and sonicated to detach adsorbed proteins. The supernatant was collected and used for protein quantification by means of the bicinchoninic acid (BCA) method (BCA Kit; Sigma-Aldrich).^31,38^

#### 2.6.8 Antimicrobial Evaluation of AMX-PDO-Treated Membranes

The antimicrobial effect of AMX-PDO membranes, compared with nontreated membranes, was evaluated in vitro using the polymicrobial biofilm model described above (item 2.2). For this purpose, stimulated human saliva from healthy volunteers was used as the microbial inoculum. Membranes were incubated with the salivary microbial inoculum in BHI medium (10:1 v/v) + sucrose (10:1 v/v) at 37 °C, with 5% CO_2_, for 24 h. Assays were conducted in duplicate. Biofilms were analyzed in terms of live cell counts.

The antimicrobial ability of the AMX-PDO membrane developed was also evaluated in situ. For this purpose, three healthy volunteers used palatal appliances containing the AMX-PDO and control membranes, which were fixed onto discs and protected by a plastic mesh to allow polymicrobial biofilm accumulation on the substrate exposed to the human oral environment. After 24 h, the membranes were collected to estimate live cell counts and microbial composition by DNA–DNA checkerboard hybridization. Live cell counts were estimated on both the membranes and the discs to evaluate whether the AMX-PDO membranes reduced the contamination of Ti discs compared with nontreated membranes.

### 2.7 Statistics

Prism 10.0 (GraphPad, Boston, USA) was used for statistical analyses and to generate the graphs. Multiple group comparisons were performed by one-way analysis of variance (ANOVA) (comparing the PDO-membranes with collagen-based membranes). Pair-wise comparisons were made with the Bonferroni *t*-test. A significance level of 5% was adopted.

## 3 Results

### 3.1 PDO-Based Membranes Presented Similar Microbial Loads when Compared with Collagen-Based Membranes

**Figure 1:**
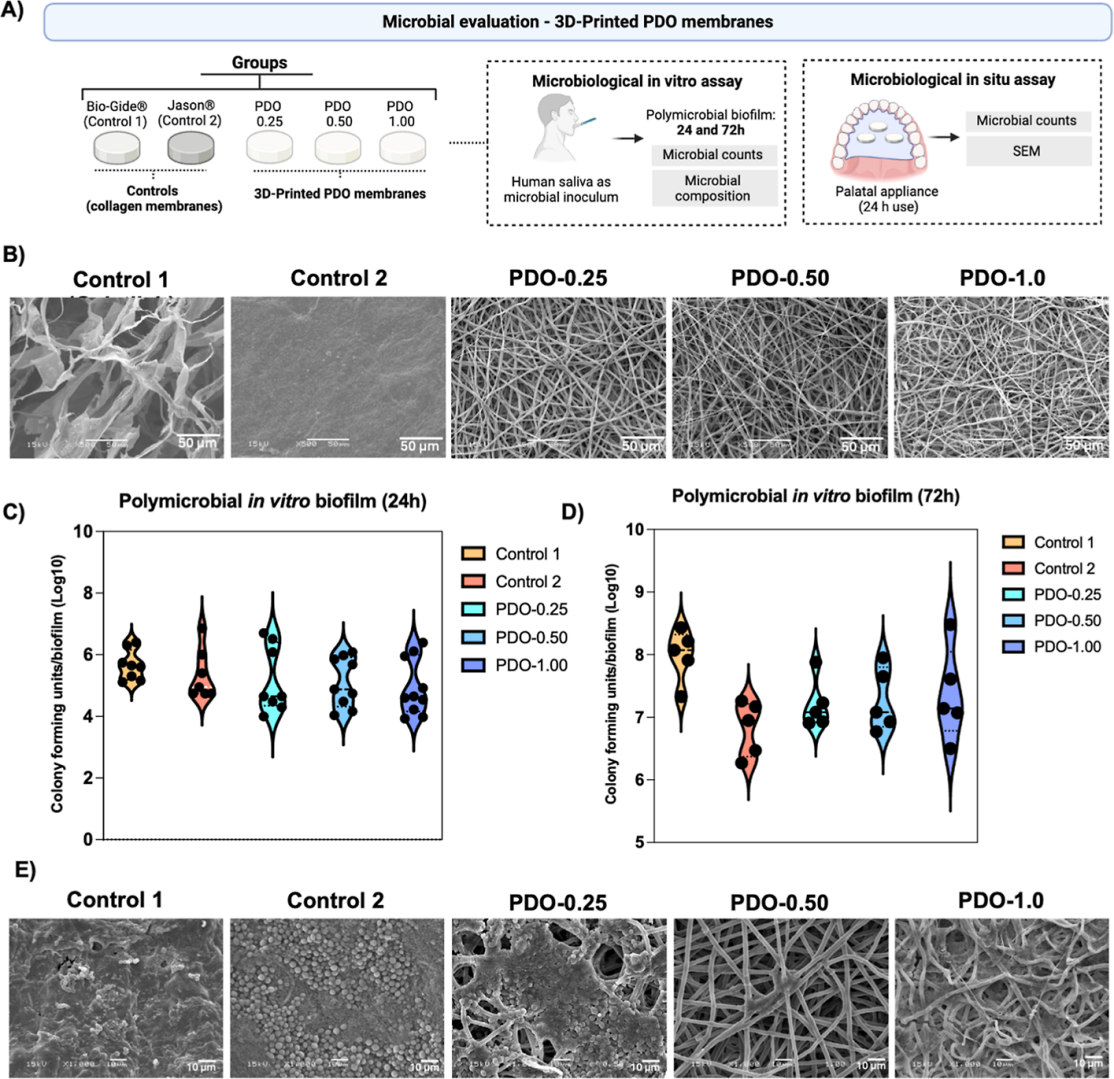
Polymicrobial contamination of polydioxanone (PDO) and collagen-based membranes. (A) Schematic diagram of the experimental design. PDO membranes with different pore sizes (0.25, 0.50, 1.00 mm) and commercially available collagen-based membranes (control 1 and 2) were evaluated for polymicrobial biofilm formation using in vitro and in situ models. Stimulated human saliva from healthy volunteers was used as the microbial inoculum to simulate the oral microbiome. (B) Scanning electron microscopy (SEM) was used to evaluate and compare membrane morphology (×500 magnification). (C) Colony-forming units (CFUs) representing total bacterial counts after 24 h of in vitro polymicrobial biofilm formation. (D) CFUs representing total bacterial counts after 72 h of in vitro polymicrobial biofilm formation (*n* = 5). (E) SEM images of membranes after 72 h of in vitro biofilm formation (×1,000 magnification).

Since previous evidence has overlooked the microbial contamination of resorbable membranes, which can affect the clinical outcomes of regenerative therapy,^21^ we first compared the polymicrobial contamination of commercially available collagen-based membranes with PDO-based membranes of different pore sizes by using in vitro and in situ models (Figure 1A). SEM images before biofilm formation showed significant differences among the PDO membranes and both controls (*p* < 0.05), as expected (Figure 1B). Collagen-based membranes exhibited a dense structure, characterized by thick, regularly shaped strips (control 1) or a solid and dense structure (control 2). In contrast, the PDO membrane displayed thin strips with irregular orientations but of consistent thickness, while the strip thickness decreased as the pore size increased. Importantly, PDO-based membranes in all pore sizes exhibited similar (*p* > 0.05) microbial counts at 24- (Figure 1C) and 72- (Figure 1D) hours of polymicrobial biofilm formation compared with collagen-based membranes. This finding was confirmed by SEM images after 72 h of biofilm formation, which showed microbial clusters on all membranes, with a predominance of coccoid-shaped microbial species intertwined within the membrane strips (Figure 1E).

**Figure 2:**
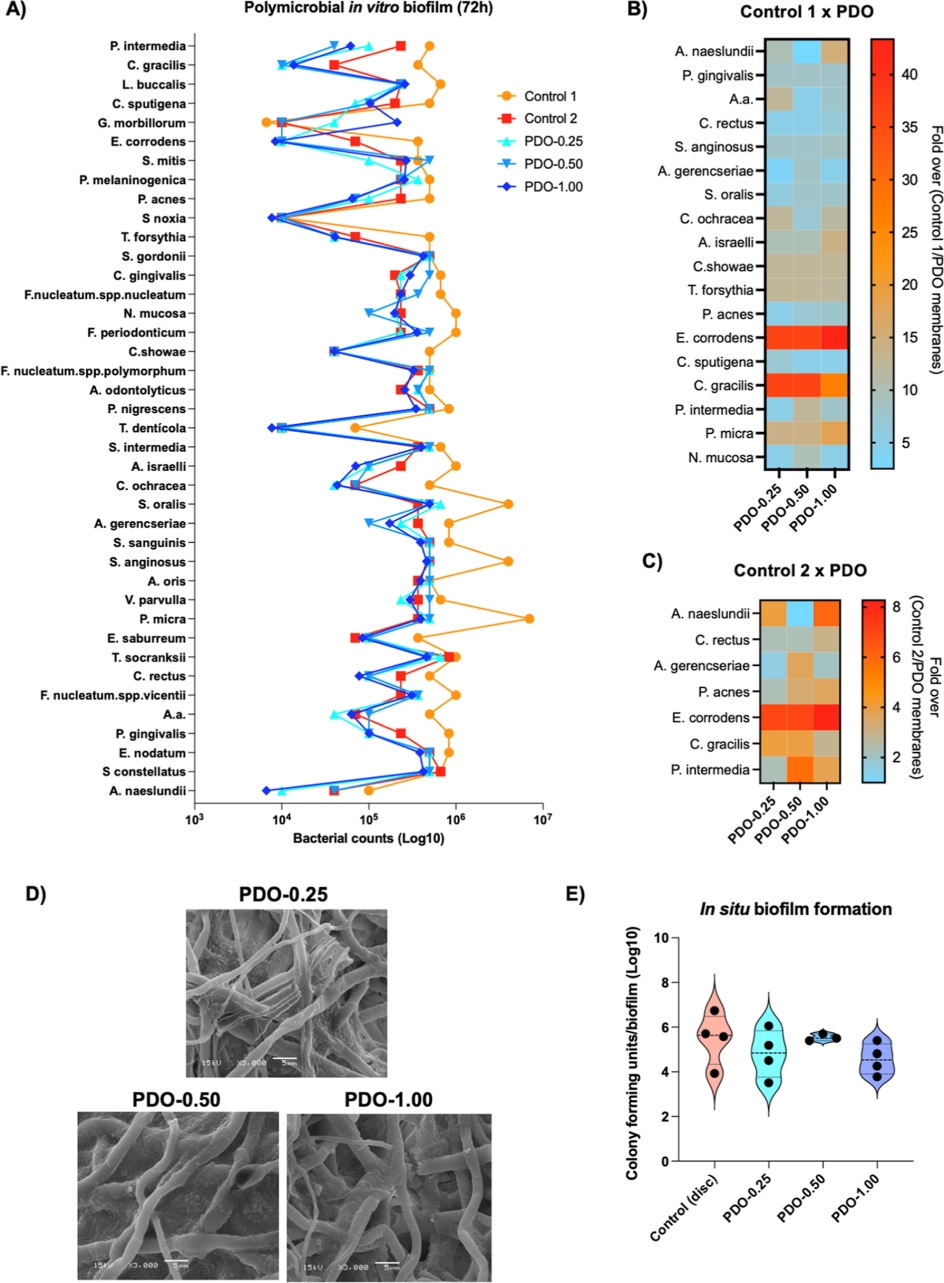
Microbial composition of polymicrobial biofilms formed in vitro on membrane structures, and in situ evaluation. (A) Levels (log10) of 40 bacterial species associated with oral infections, evaluated using DNA–DNA checkerboard hybridization. (B) Fold-change (control 1/PDO) of bacterial levels showing at least a 5-fold higher count for control 1 compared with PDO membranes, with an increase observed in at least one pore size type of PDO membrane. (C) Fold-change (control 1/PDO) of bacterial levels showing at least a 3-fold higher count for control 2 compared with PDO membranes, with an increase observed in at least one pore size type of PDO membrane. (D) Scanning electron microscopy (SEM) images (×3,000 magnification) of PDO membranes with different pore sizes after in situ biofilm formation. Membrane discs placed on titanium discs were inserted into palatal appliances in the oral cavities of healthy volunteers and worn for 3 days. (E) Colony-forming units (CFUs) of total bacterial counts after in situ polymicrobial biofilm formation on PDO membranes. Titanium discs not covered by membranes were used as controls. Error bars indicate standard deviations.

Although total bacterial counts were similar among the tested membranes, the composition and structure of the membranes, however, modulated the microbial composition of biofilms (Figure 2A). When compared with collagen-based membranes, PDO membranes demonstrated reduced levels of important oral pathogens. Eighteen bacterial species exhibited levels at least 5× higher on control 1 (collagen-based) than on at least one of the PDO membrane pore sizes (Figure 2B), including key putative pathogens such as *Porphyromonas gingivalis* and *Tannerella forsythia*. Furthermore, seven bacterial species showed at least 3× higher counts on control 2 compared with PDO membranes in at least one of the pore sizes tested (Figure 2C), including Actinomyces and Prevotella species. Therefore, although the total live bacterial counts were similar, PDO-based membranes exhibited reduced levels of bacteria associated with oral infections when compared with collagen-based membranes. Importantly, among the PDO membranes with different pore sizes, the microbial composition was similar, with comparable bacterial levels (Figure S1–microbial composition on the PDO membranes in different sizes).

When the PDO membranes with different pore sizes were compared among them, our in situ model, in which membranes were inserted into the oral cavities of volunteers, showed similar patterns of biofilm clusters impregnated into the membranes, as observed in SEM images (Figure 2D), as well as similar live bacterial counts across all pore sizes (Figure 2E). Thus, the pore size of PDO membranes does not significantly modulate microbial accumulation.

### 3.2 Amoxicillin Loading on PDO Membranes Using Plasma Technology did not Affect the Physical, Mechanical, or Biological Properties of the Material

**Figure 3:**
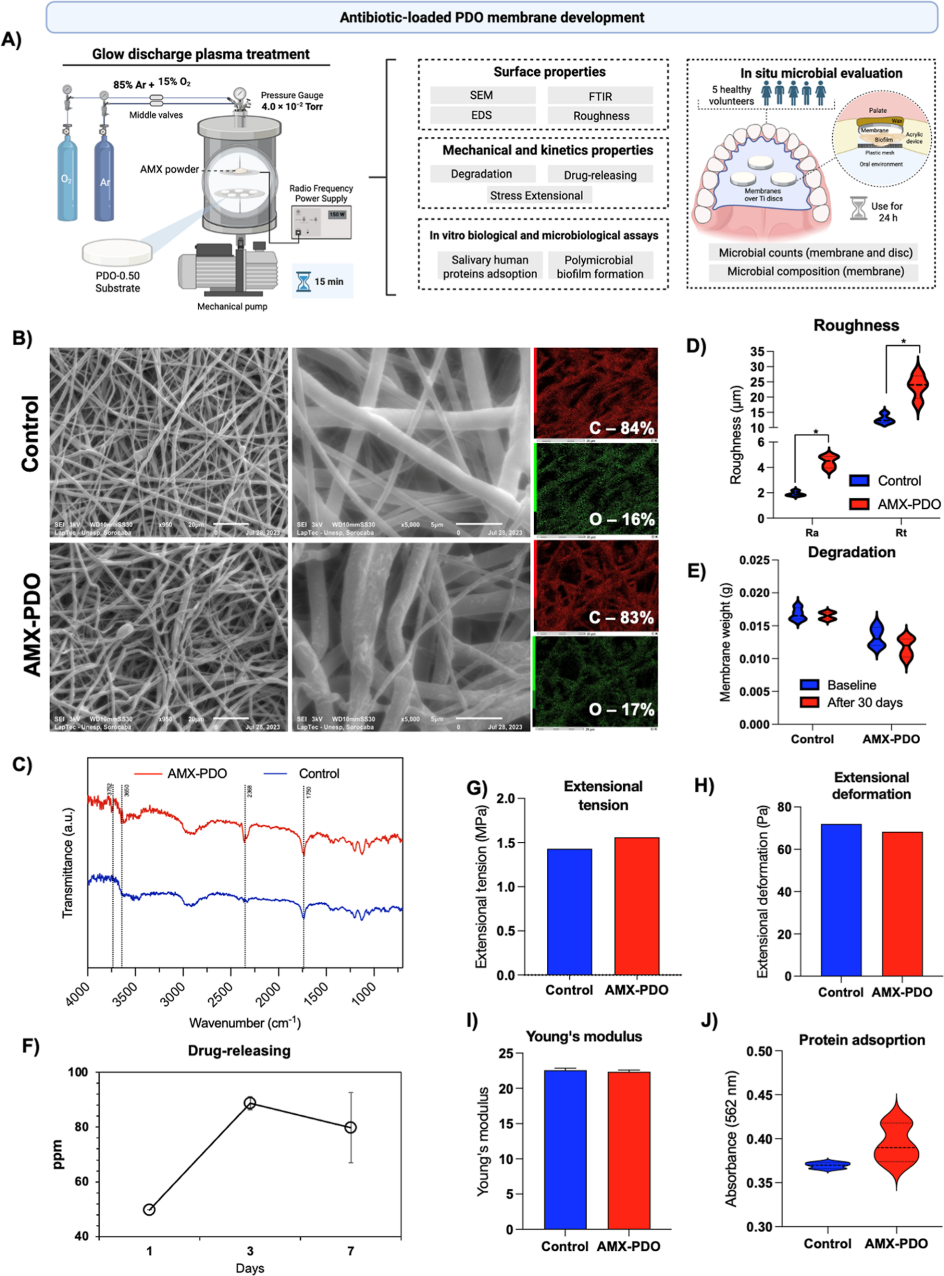
Development of antibiotic-loaded PDO membranes. (A) Schematic diagram of the experimental design. Amoxicillin-loaded PDO-0.50 membranes (AMX-PDO) were developed using glow discharge plasma technology. The membranes developed were evaluated for physical, mechanical, biological, and microbiological properties. Nontreated PDO-0.50 membranes were used as controls. (B) Scanning electron microscopy (SEM) images of AMX-PDO and control membranes, in addition to EDS analysis for chemical composition. (C) FTIR analysis to determine the molecular structure of the treatment based on the peaks. (D) Membrane surface roughness evaluated using a profilometer, estimating average roughness (Ra) and total roughness (*R*_t_) in μm. (E) Degradation of membranes after immersion in 0.9% NaCl and incubation for 30 days at 37 °C. Membrane weights were measured before and after the assay. (F) Amoxicillin release assay evaluated by HPLC and expressed as ppm. Membranes were immersed in NaCl solution and incubated for 1, 3, and 7 days, and the supernatant was analyzed to estimate antibiotic concentration. (G) Tensile resistance measuring extensional tension resistance, (H) extensional deformation resistance, and (I) Young’s modulus. (J) Human salivary protein adsorption on membranes after 2 h of incubation, evaluated using the bicinchoninic acid (BCA) method and expressed as absorbance at 562 nm. *p < 0.05, using the Bonferroni *t*-test. Error bars indicate standard deviations.

Glow discharge plasma treatment was used to load the antibiotic amoxicillin onto PDO-0.50 membranes, a commonly used pore size for resorbable membranes in GBR^22^ (Figure 3A). As expected, the combination of gases and antibiotics slightly altered the surface morphology of the membrane mesh. SEM images revealed globular structures adhered to the membrane strips, possibly representing antibiotic deposition, and a more irregular strip morphology compared with the control (Figure 3B). Although the basic chemical composition of the membranes was not affected, as indicated by EDS analysis (Figure 3B), FTIR analysis showed specific peaks, suggesting the presence of amoxicillin on the membrane structure, as evidenced by the presence of molecular linkages of C–H (2368 cm^–1^), C=O (1750 cm^–1^), C–H–N (3650 cm^–1^) and O–H (3752 cm^–1^) (Figure 3C). The slight change in the membrane structure significantly increased (*p* < 0.05) the those of the roughness, as indicated by the *R*_a_ and *R*_t_ results, with an average *R*_a_ of 4.44 μm (±0.48) (Figure 3D). Biodegradability is an important outcome for resorbable membranes. The antibiotic loading on PDO membranes did not alter the degradation (weight loss) of the AMX-PDO membranes after 30 days of incubation in solution, showing similar results to those of the control group (Figure 3E). The antibiotic loaded onto the membrane needs to be released into the environment to act as an antimicrobial agent to control local infection. The drug-release assay confirmed that the drug was loaded onto the membrane structure and that 49.8 ppm of AMX was released, equivalent to 49.8 μg/mL, after 24 h (Figure 3F). The concentration showed an approximately a 2-fold increase to 88.75 ppm after 3 days and remained stable at 79.5 ppm after 7 days, indicating that a concentration close to 80 ppm represents the maximum release of the antibiotic by the treated membrane over time.

One of the main advantages of synthetic membranes is their good space-maintenance ability and adaptability to bone defects. Therefore, resistance to tension and deformation is an important outcome to maintain after plasma treatments. The AMX-PDO membrane showed no difference in extensional tension resistance (Figure 3G), extensional deformation resistance (Figure 3H), and Young’s modulus compared with the control nontreated membranes. To confirm the basic properties of the membrane developed, the biological response was evaluated. For this purpose, a protein adsorption assay was conducted, as it is considered the first biological response in the human body, which mediates subsequent biological processes. The AMX-PDO membrane showed similar human saliva-adsorbed proteins compared with the control (*p* > 0.05) (Figure 3J).

### 3.3 AMX-PDO Membranes Reduced Microbial Accumulation and the Passage of Microbial Species through the Membrane Structures, while also Positively Modulating Biofilm Composition

**Figure 4:**
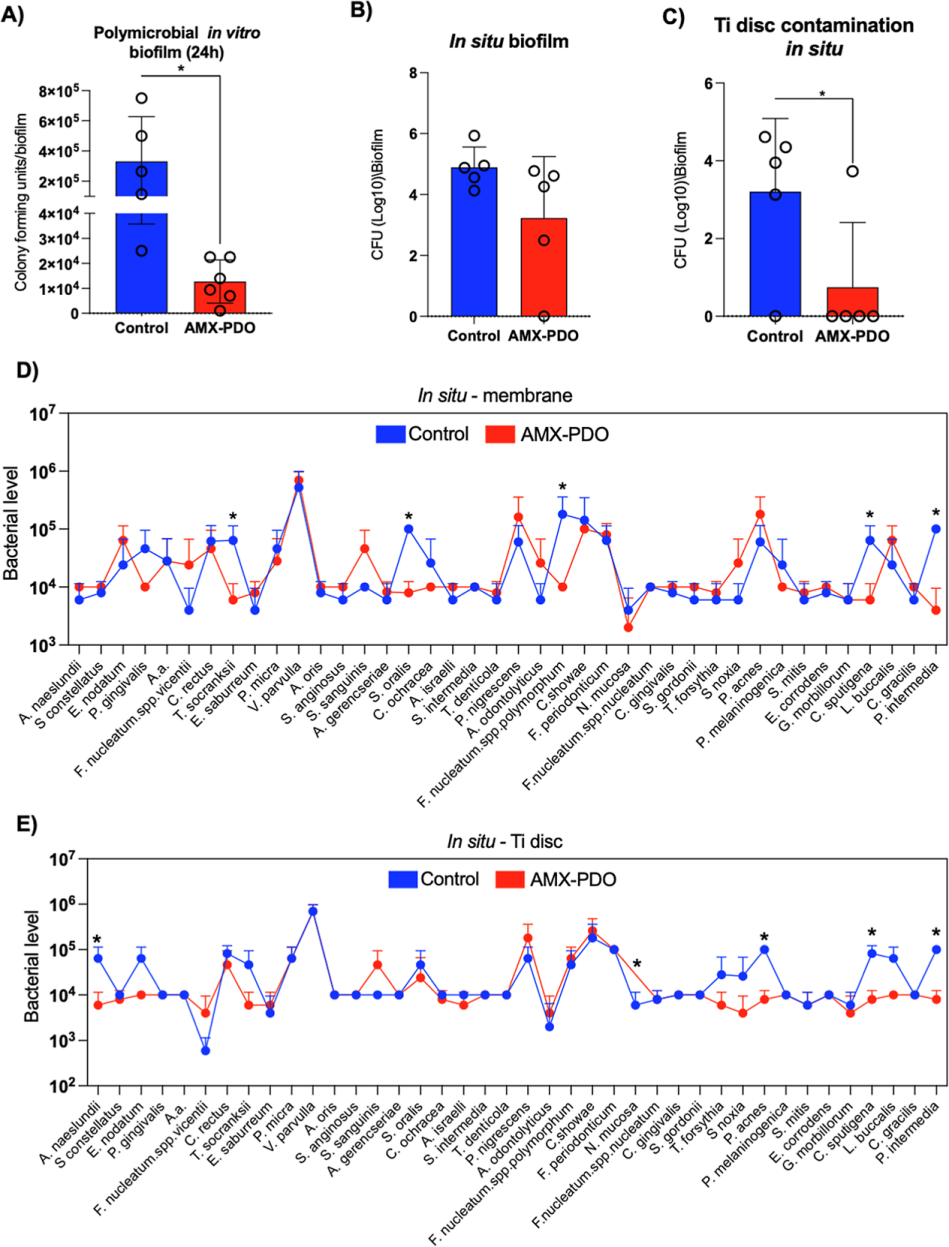
Antimicrobial evaluation of amoxicillin-loaded (AMX-PDO) membranes when compared with the control. (A) Colony-forming units (CFUs) of total bacterial counts after 24 h of in vitro polymicrobial biofilm formation on PDO membranes. (B) CFUs of total bacterial counts after 24 h of in situ polymicrobial biofilm formation on PDO membranes. (C) CFUs of total bacteria counts after 24 h of in situ polymicrobial biofilm formation on titanium discs placed under PDO membranes. (D) Levels (log10) of 40 bacterial species associated with oral infections on PDO membranes, evaluated using DNA–DNA checkerboard hybridization after in situ biofilm formation. (E) Levels (log10) of 40 bacterial species associated with oral infections on titanium discs placed on PDO membranes, evaluated using DNA–DNA checkerboard hybridization after in situ biofilm formation. *p < 0.05, using the Bonferroni *t*-test. Error bars indicate standard deviations. Error bars indicate standard deviations.

AMX-PDO membranes significantly (*p* < 0.05) reduced in vitro polymicrobial 24 h biofilm formation on the membrane structure (Figure 4A) in comparison with nontreated membranes, showing a 10-fold (1-log) reduction in total bacterial counts. Although not statistically significant, a slight reduction was observed in our in situ model, with lower levels of bacterial counts on AMX-PDO membranes (Figure 4B). Importantly, antibiotic loading onto the membrane structure reduced the passage of microbial species to the implant surface in the in situ model, as Ti discs placed on AMX-PDO membranes showed significantly (*p* < 0.05) reduced bacterial counts when compared with nontreated membranes (Figure 4C).

AMX-PDO membranes led to a significant reduction in five bacterial species associated with the progression of peri-implant infections, compared with control: *Treponema socranskii*, *Streptococcus oralis*, *Fusobacterium nucleatum subsp*. *polymorphum*, *Capnocytophaga sutegana*, and *Prevotella intermedia* (Figure 4D). The reduction in microbial accumulation on AMX-PDO membranes also modulated the contamination of Ti discs placed on the membranes, with the AMX-PDO group showing a reduction in five bacterial species: *Actinomyces naeslundii*, *Neisseria mucosa*, *Propionibacterium acnes*, *C. sutegana*, and *P. intermedia* (Figure 4E). Notably, two species were reduced on the membranes—*C. sutegana* and *P. intermedia*—were also reduced on the Ti discs.

## 4 Discussion

Microbial contamination of wounds and barrier membranes are clinical challenges that often lead to treatment failures.^21^ Our findings showed similar microbial counts for both PDO and collagen-based membranes. However, in terms of microbial composition, which plays a key role in triggering inflammatory processes, PDO membranes demonstrated reduced levels of important putative oral pathogens, when compared with collagen membranes. This could be due to their protein-rich composition, which serves as a potential source of nutrients for proteolytic bacteria. Bacteria such as *P. gingivalis* and *F. nucleatum*, are known to thrive in environments where proteins are present, particularly collagen and other extracellular matrix components.^39^ Importantly, the pore sizes of PDO membranes did not affect in situ biofilm formation, showing similar outcomes when the membranes were inserted into the oral cavity of volunteers. To address microbial contamination on PDO membranes, we developed an antibiotic-loaded PDO membrane using low-pressure glow discharge plasma technology, which is commonly applied to biomedical Ti implants.^34^ The antibiotic-loaded membranes significantly reduced polymicrobial accumulation on the membranes and prevented microbial cells from passing through to contaminate implantable devices. Therefore, the AMX-PDO membranes developed retained the barrier membrane properties expected, particularly since we used a commercially available membrane, while introducing a new and important feature: microbial control, which may help prevent initial infections. Thus, our antibiotic-loaded membranes function as more than passive physical barriers only. These membranes actively mitigate microbial colonization and reduce the risk of contamination, making them a more protective and biologically active barrier. Their dual role enhances the regenerative environment by not only shielding the surgical site mechanically but also exerting antimicrobial activity, which reduces inflammation and infection risks. This biologically active feature elevates their protective efficacy compared with traditional membranes, fostering improved outcomes in tissue regeneration.

Synthetic absorbable polymers have been widely used for manufacturing barrier membranes, due to their excellent uniformity in production, favorable physical-mechanical properties, and nontoxicity, as their hydrolysis generally does not induce immune responses.^40^ PDO-based membranes have been used for orbital floor fracture,^41^ defects in the femoral head,^42^ and critical bone defects.^43^ Importantly, PDO polymer has a lower degradation rate compared with other polymers, being considered a slow to moderately degrading polymer that loses its mass within 6–12 months through hydrolytic degradation.^44^ Previous in vivo evidence has compared PDO and collagen-based commercially available membranes and have shown no difference in terms of the percentage of connective tissue, newformed bone, and remaining biomaterial.^44^ Moreover, both membranes used in our study (collagen and PDO) have shown no systemic toxicity and the ability to induce cell migration, adhesion, spreading, and proliferation.^20^ Thus, both membranes, which are commercially available at present, have pivotal properties for use during GBR in clinical practice. However, microbial contamination continues to be a concern.

Recently, an in vitro study evaluated the microbial contamination of the same PDO membranes as those used in this study, compared with collagen-based types, and showed reduced microbial loads for PDO.^45^ Although it was a multispecies biofilm, the study did, however, use specific microbial species, which did not represent the entire oral microbiome, in the same way as we tested it, using human saliva as the microbial inoculum. Here, we found similar total microbial counts for both collagen membranes and the three PDO membranes with different pore sizes. However, *P. gingivalis* and *T. forsythia* showed more than a 5-fold reduction on PDO membranes compared with collagen-based membranes (control 1). These bacterial species are considered the main contributors to implant-related infections and periodontal tissue destruction.^46^ Moreover, *P. gingivalis* has demonstrated significant inflammatory induction during regenerative therapy using mesenchymal stem cells.^47^ Additionally, collagen-binding proteins are recognized for promoting microbial adhesion, including that of *P. gingivalis* and *T. forsythia*.^39^ Importantly, in this study, we considered microbial presence throughout the entire membrane, not on its surface only, as microbial cells can become trapped within the membrane structure. Further studies are needed to explore the distribution and dispersion of these microbial species throughout the membrane structure and to evaluate a broader range of microbial species.

To address polymicrobial accumulation, we developed an amoxicillin-loaded PDO membrane with the aim of preventing and controlling local infections during regenerative therapy. Bioengineering technologies have been widely applied to develop barrier membranes with antimicrobial properties, as this ability is of utmost importance, with acute infection being identified as one of the main complications during GBR.^21,48,49^ Although antibiotics have been one of the main agents loaded onto barrier membranes, mainly using electrospinning technology, various other antimicrobial agents, such as silver, zinc, and chlorhexidine, have also been incorporated into membranes.^21,50^ The rationale for using antibiotics loaded onto membranes is based on their broad-spectrum effects, targeting both Gram-positive and Gram-negative bacterial species. Moreover, systemic antibiotics such as metronidazole and amoxicillin have been the primary choices as adjunctive treatments for periodontal disease and implant-related infections, and have effectiveness against oral bacteria species.^51,52^ Although clinical evidence has shown no additional benefit of systemic antibiotics during GBR, the use of amoxicillin, a broad-spectrum antibiotic, has demonstrated some effectiveness in controlling initial inflammation during GTR.^53,54^ This antibiotic is highly recognized for its effectiveness against the oral microbiome, drastically reducing the metabolic activity of tooth-related biofilms.^55^ Therefore, since the prolonged use of antibiotic protocols, including amoxicillin, leads to an increased risk of antimicrobial resistance^56^ and systemic side effects, the development of local therapies is of utmost importance for clinical practice.

Importantly, both materials (PDO-based membranes and the amoxicillin antibiotic) used in this study are commercially available at present and have been used in dental clinical practice in an isolated form. The PDO membranes tested in our study are commercially available and have previously been tested in in vivo models, with no toxicity effects expected.^20^ Moreover, amoxicillin is an antibiotic widely used in dental practice. The antibiotic concentration included in the reactor during plasma treatment (1 g) is the same concentration as that commonly used daily to treat periodontal infections in dental practice, which recommends this regimen for 14 days.^51^ Importantly, as shown by the release assay, approximately 10% of the concentration was incorporated into the membrane structure. Systemic use of amoxicillin results in a concentration of 14.05 μg/mL in the gingival crevicular fluid,^57^ which has shown effectiveness against oral biofilm-induced diseases. Here, we achieved a release concentration three times higher within 24 h, which remains below the toxicity threshold for human osteoblasts and cell lines.^58^ Moreover, the protein adsorption level observed for AMX-PDO membranes—an important step mediating subsequent biological responses^59^—suggests the maintenance of biocompatibility properties. However, this aspect requires further exploration, particularly concerning bone-related cells.

Glow discharge plasma technology have been widely used to create coatings with antimicrobial abilities and improved biological responses on biomedical devices.^34,60^ Since we used commercially available PDO membranes, it was important to preserve their physical-mechanical properties to maintain their established clinical applications. The plasma treatment did not alter membrane morphology, biodegradability, or tensile resistance, which are important aspects for GBR therapy. Although roughness was increased, it is unlikely to affect biological behavior, given the inherently irregular structure of synthetic barrier membranes.

Previous studies, using encapsulated amoxicillin in poly(D,l-lactic acid) (PDLLA) nanofiber membranes for GTR applied by the electrospinning technique, showed no changes in the membrane morphology, 60% antibiotic release within the first week, and a reduced inflammatory response.^26^ The antimicrobial effect against two bacterial species: *Streptococcus sanguinis* and *Porphyromonas gingivalis*, was evaluated and reported.^26^ However, oral infections have a polymicrobial profile,^5^ and the development of new biomaterials needs to address this characteristic. The functionalization of amoxicillin on PDO membranes has not previously been reported in the literature. However, PDO polymer has been highlighted as a promising agent for sustained drug delivery, mainly through copolymerization, encapsulation of agents, and electrospinning, with the effectiveness being highly related to the hydrophobicity of the polymer.^61^

Antibiotic resistance is a common concern relative to the use of such drugs loaded onto biomaterials. Importantly, the use of amoxicillin (500 mg) twice a day for 14 days has been the main treatment protocol for periodontal disease and has been widely used clinically for more than a decade.^51^ Clinical evidence has shown that the use of a higher dosage of amoxicillin (1 g, three times a day) led to a modest and short-lived increase in amoxicillin-resistant bacteria.^62^ Moreover, important oral bacteria are susceptible to the effects of amoxicillin, leading to bacterial death,^63^ and the concentration released from our membrane is more than five times higher than the minimum inhibitory concentration previously reported for common periodontal pathogens.^64^ Therefore, it is expected that the antibiotic released here does not lead to antimicrobial resistance, especially considering that the entire antibiotic content was released within 3–7 days. However, this outcome requires further molecular evaluation. Another important consideration is the potential for the antibiotic to induce microbial changes, such as an effect of dysbiosis. Nevertheless, given the rapid antibiotic release, its concentration, and the microbial findings showing a reduction in important pathogens, it is expected that the antibiotic-loaded membranes will promote a health-associated profile.

Our newly developed AMX-PDO membranes were able to reduce approximately 1.3-log of in vitro polymicrobial biofilm. This showed that antibiotic released was able to control microbial accumulation or lead to bacteriostatic and/or bactericidal effects. The antimicrobial effect may prevent initial infections during wound healing, which can be applied to other mucosal membranes and extra oral sites. One of the main purposes of barrier membranes is to preserve cellular components at the wound site and prevent the passage of external agents that affect wound healing and the regenerative process.^8,10^ Therefore, antimicrobial ability is crucial to prevent the passage of viable microbial cells, transforming a simply “physical barrier membrane” into a biologically active membrane. In fact, our in situ findings showed reduced live microbial counts on the implant surface placed above the AMX-PDO membrane, demonstrating its effectiveness in reducing wound contamination. *P. intermedia* and *F. nucleatum* microbial species were significantly reduced on the AMX-PDO membranes. These bacteria are highly associated with periodontal diseases and implant-related infections.^65,66^*P. intermedia* was also significantly reduced on the implant surface, demonstrating the effectiveness of AMX-PDO membranes in reducing the risk of infections. Amoxicillin demonstrates an effective antimicrobial action against a wide range of Gram-positive bacteria and some Gram-negative species, acting by binding to penicillin-binding proteins, which results in cell wall lysis and bacterial cell destruction.^67^ Therefore, the functionalization of amoxicillin on PDO membranes effectively reduced microbial loads and modulated biofilm composition. Further studies using high-throughput techniques are needed to evaluate the antimicrobial effect across a wide range of microbial species and at different stages of microbial contamination to fully understand its effectiveness.

In addition to its potential applications in oral tissue regeneration, the AMX-PDO membrane, with its antibiotic-loaded and functionalized properties, could prove valuable in a variety of other medical fields. Its ability to reduce microbial biofilm and prevent infection makes it ideal for use in other mucosal sites, such as the nasal passages, gastrointestinal tract, or any other mucosal sites, where bacterial contamination can impede healing and lead to further complications. Furthermore, the membrane could be beneficial in treating chronic skin ulcers, including diabetic foot ulcers and pressure sores, where infection control and wound healing are critical. By offering a personalized, electrospinning fabrication, the AMX-PDO membrane can be tailored to the specific size and shape of the wound site, improving its fit and effectiveness in treating complex or irregularly shaped wounds. The antimicrobial properties of the membrane also offer a significant advantage in preventing infection at these sites, reducing the need for systemic antibiotics and promoting more localized, controlled healing. Beyond wound care, this type of functionalized membrane could be used in surgical procedures that require tissue regeneration, such as soft tissue grafting or implant surgeries, where preventing microbial contamination is essential for long-term success.^68^ The adaptability of the AMX-PDO membrane, combined with its biologically active properties, holds great promise for a broad range of medical applications, from dermatology to implantology and other fields.

## 5 Conclusion

Thus, PDO-based membranes exhibited a similar load of polymicrobial accumulation to those of commercially available collagen-based membranes. However, PDO membranes were able to modulate oral microbial communities, significantly reducing the levels of proteolytic oral pathogens associated with biofilm-induced diseases. To control microbial accumulation, amoxicillin was successfully loaded onto the PDO membrane structure by plasma treatment, without altering the physical or mechanical properties of the membrane. The antibiotic released from the treated-membrane effectively reduced polymicrobial accumulation, preventing viable microbial cells from passing through the membrane and contaminating implant devices placed above it. This antimicrobial effect resulted in a decrease in both total bacterial levels and specific microbial species, including those related to periodontal disease and implant-related infections.
