# Supplementary material for: Tackling Microbial Contamination in Polydioxanone-Based Membranes for Regenerative Therapy: Bioengineering an Antibiotic-Loaded Platform
Source: ACS Appl Bio Mater. 2025 Apr 30;8(5):4119–31. doi: 10.1021/acsabm.5c00263 (PMC12093370; doi:10.1021/acsabm.5c00263)

SUPPORT INFORMATION

Tackling microbial contamination in polydioxanone-based membranes for regenerative therapy: bioengineering an antibiotic-loaded platform

Victoria L Abdo[1], Jamil A Shibli[1], Raphael C. Costa[2], Maria H. Rossy Borges[3], Ademar Wong[4], Maria D. P. T. Sotomayor[4], Martinna Bertolini[5], Luciene C. Figueiredo[1], Valentim A. R. Barão[3], Elidiane C. Rangel[6], Joao Gabriel S. Souza[1]*

[1] Dental Research Division, Universidade Universus Veritas Guarulhos, Guarulhos, 07023-070, Brazil.

[2] School of Dentistry, Alfena's Federal University (UNIFAL-MG), Alfenas, 37130-001, Brazil.

[3] Departamento of Prosthodontics and Periodontology, Piracicaba Dental School, Universidade Estadual de Campinas (UNICAMP), Piracicaba, 13414-903, Brazil.

[4] Institute of Chemistry, State University of São Paulo (UNESP), Araraquara, 14801-970, Brazil

[5] Department of Periodontics and Preventive Dentistry, University of Pittsburgh School of Dental Medicine, Pittsburgh, PA, 15213, USA.

[6] Laboratory of Technological Plasmas, Institute of Science and Technology, São Paulo State University (UNESP), Sorocaba, 18087-180, Brazil.

Corresponding Author

* João G. S. Souza, Department of Periodontology, Dental Research Division, Universidade Universus Veritas Guarulhos, Guarulhos, São Paulo 07023-070, Brazil.

E-mail: jgabriel.ssouza@yahoo.com.br

Figure S1 - Microbial composition of polymicrobial biofilms formed in vitro on membrane structures. Levels (log10) of 40 bacterial species associated with oral infections, evaluated using DNA-DNA checkerboard hybridization according to PDO membranes pore sizes.

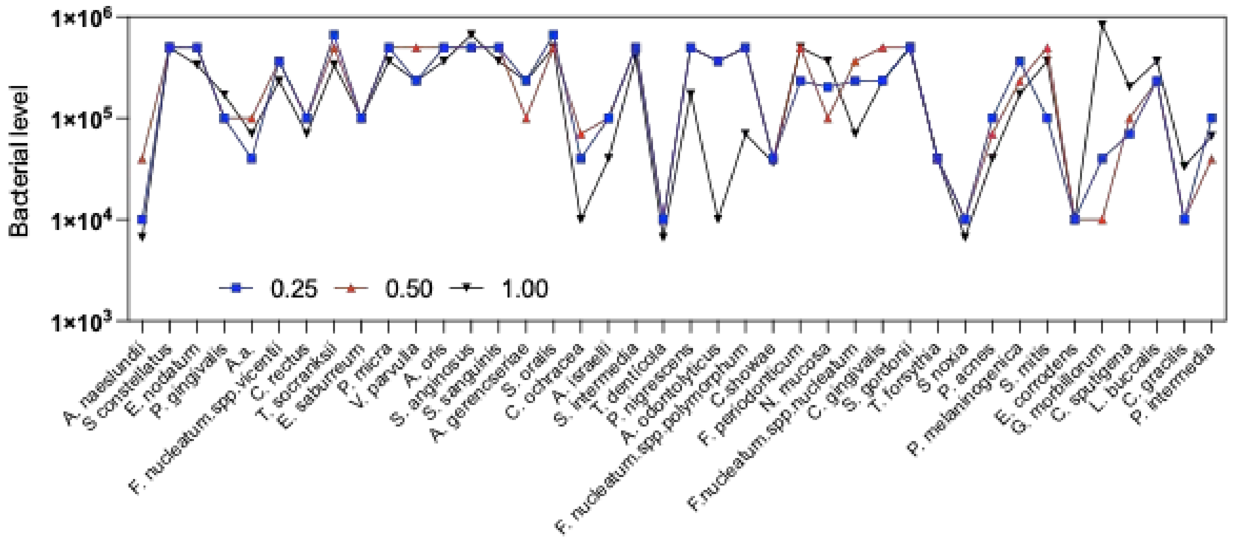

Supplement: Supplementary file 1 — mt5c00263_si_001.pdf [file mt5c00263_si_001.pdf]
